# Glucagon-Like Peptide-1 Receptor Agonists Across the Heart Failure Spectrum: A Systematic Review and Meta-Analysis

**DOI:** 10.7759/cureus.110621

**Published:** 2026-06-10

**Authors:** Vicky Muller Ferreira, Victor Ayres Muller

**Affiliations:** 1 Cardiology, Independent Research, Rio de Janeiro, BRA; 2 Internal Medicine, Hospital Universitario De Vassouras, Rio de Janeiro, BRA

**Keywords:** cardiovascular outcomes, glucagon-like peptide-1 receptor agonists, heart failure, hfpef, meta-analysis, semaglutide, systematic review, tirzepatide

## Abstract

Glucagon-like peptide-1 receptor agonists (GLP-1 RAs) have shown cardiovascular (CV) benefits, but their effects across the heart failure (HF) spectrum remain uncertain. We synthesized randomized evidence comparing GLP-1 RAs with placebo in adults with HF across ejection fraction phenotypes by searching PubMed, Cochrane CENTRAL, and ClinicalTrials.gov through February 2026. The primary outcome was the composite of CV death and first HF hospitalization, and random-effects meta-analysis used restricted maximum likelihood (REML) estimation with the Hartung-Knapp-Sidik-Jonkman (HKSJ) adjustment. We included 14 studies (six dedicated HF trials and eight cardiovascular outcomes trials (CVOT) HF subgroup analyses) encompassing 18,184 patients. The primary composite outcome was not statistically significant (hazard ratio (HR) 0.86, 95% confidence interval (CI) 0.73-1.01; P=0.067; I²=47%). GLP-1 RAs reduced all-cause mortality (ACM; HR 0.86, 95% CI 0.79-0.95; P=0.008; low certainty according to Grading of Recommendations, Assessment, Development, and Evaluations (GRADE)) and major adverse CV events (MACE; HR 0.80, 95% CI 0.69-0.93), and improved Kansas City Cardiomyopathy Questionnaire Clinical Summary Score (KCCQ-CSS) by 7.4 points and 6-minute walk distance (6MWD) by 17.6 m. However, the mortality benefit was driven by CVOT subgroups, whereas dedicated HF trials showed directional harm. GLP-1 RAs did not significantly reduce the primary composite outcome but improved quality of life and functional capacity in heart failure with preserved ejection fraction (HFpEF) and obesity. The pooled mortality reduction should be interpreted cautiously, given the divergence between indirect and direct evidence.

## Introduction and background

Heart failure (HF) remains a major global public health challenge, affecting over 64 million people worldwide and imposing substantial morbidity, mortality, and healthcare costs [1]. Despite advances in neurohormonal blockade for heart failure with reduced ejection fraction (HFrEF), therapeutic options for heart failure with preserved ejection fraction (HFpEF) have been historically limited [2,3]. The recognition that obesity and metabolic dysfunction are central to HFpEF pathophysiology has opened new avenues for treatment [4].

Glucagon-like peptide-1 receptor agonists (GLP-1 RAs), initially developed for type 2 diabetes mellitus (T2DM), have demonstrated robust cardiovascular (CV) benefits beyond glucose lowering [5]. These agents reduce body weight, improve insulin sensitivity, decrease systemic and vascular inflammation, lower blood pressure, and may exert direct cardioprotective effects through multiple pathways [6]. HF-relevant mechanisms include epicardial fat reduction, modest natriuresis, improved myocardial energetics, and weight-loss-mediated effects on ventricular geometry [6]. Large CV outcomes trials (CVOTs) and meta-analyses have established the CV efficacy of several GLP-1 RAs in high-risk cardiometabolic populations [7,8].

Recently, dedicated HF trials have provided pivotal evidence. The STEP-HFpEF and STEP-HFpEF-DM trials demonstrated that semaglutide 2.4 mg significantly improved symptoms, functional capacity, and weight in patients with HFpEF and obesity [9,10]. The SUMMIT trial showed that tirzepatide reduced the composite of CV death or worsening HF events in HFpEF with obesity [11]. Conversely, the FIGHT trial of liraglutide in patients recently hospitalized for HFrEF did not demonstrate clinical benefit [12].

Prior meta-analyses of GLP-1 RAs in CV and renal populations preceded several landmark dedicated HF trials [7]. Recent reviews by Behers et al. and Zhang et al. have begun to incorporate newer trials, but no synthesis to date has simultaneously included SUMMIT, the SELECT HF subgroup, the SOUL HF analysis, and the FLOW HF subgroup data alongside CVOT subgroups across all HF phenotypes, or explicitly addressed the divergent mortality signals between indirect (CVOT subgroup) and direct (dedicated HF trial) evidence [13,14]. Combining both evidence streams is methodologically justified: dedicated HF trials provide direct but limited evidence (small k, few adjudicated event counts), while CVOT HF subgroups provide indirect but larger evidence; triangulating them under a pre-specified subgroup by study type allows formal heterogeneity testing of class effect across evidence strengths.

We therefore conducted a prospectively registered systematic review and meta-analysis, Prospective Register of Systematic Reviews (PROSPERO; CRD420261299844) of randomized evidence for GLP-1 RAs versus placebo in adults with HF across all ejection fraction phenotypes. Our prespecified aims were (a) to quantify pooled effects on the primary composite of CV death and first HF hospitalization and on prespecified secondary efficacy, functional, patient-reported, and safety outcomes; (b) to test heterogeneity by HF phenotype (HFpEF vs. HFrEF vs. mixed), GLP-1 RA agent, diabetes status, and study type (dedicated HF trial vs. CVOT HF subgroup); and (c) to reconcile divergent mortality signals between indirect (CVOT subgroup) and direct (dedicated HF trial) evidence using complementary sensitivity, leave-one-out (LOO), and trial sequential analyses with formal certainty assessment by Grading of Recommendations, Assessment, Development, and Evaluations (GRADE).

## Review

Methodology

Protocol and Registration

This systematic review was prospectively registered with the International PROSPERO (CRD420261299844) and conducted in accordance with the Preferred Reporting Items for Systematic Reviews and Meta-Analyses (PRISMA) 2020 guidelines [15]. Protocol amendments are documented transparently in the public PROSPERO record and in the Appendix of this Review (Appendix D).

Eligibility Criteria

We included randomized controlled trials (RCTs) that enrolled adults (age ≥18 years) with an established diagnosis of HF (any left ventricular ejection fraction (LVEF) phenotype: heart failure with reduced ejection fraction (HFrEF, ≤40%), HF with mildly reduced ejection fraction (HFmrEF, 41%-49%), or HFpEF (≥50%)), randomized to a GLP-1 RA (semaglutide, liraglutide, tirzepatide, dulaglutide, exenatide, lixisenatide, or albiglutide) versus placebo or usual care, with a minimum sample size of 50 participants and follow-up ≥12 weeks, reporting at least one outcome of interest. We included both dedicated HF trials and pre-specified or post-hoc HF subgroup analyses from cardiovascular outcomes trials (CVOTs) when HF-specific data were separately reported. Exclusion criteria included type 1 diabetes mellitus as the primary indication, observational studies, single-arm trials, case reports/series, conference abstracts without full publications, and animal or in vitro studies. The full inclusion and exclusion criteria are presented in Table 1.

**Table 1:** Eligibility criteria Inclusion and exclusion criteria were applied during study selection. PICO framework with one criterion per row. RCT, randomized controlled trial; HF, heart failure; LVEF, left ventricular ejection fraction; HFrEF, heart failure with reduced ejection fraction; HFmrEF, heart failure with mildly reduced ejection fraction; HFpEF, heart failure with preserved ejection fraction; GLP-1 RA, glucagon-like peptide-1 receptor agonist; T1DM, type 1 diabetes mellitus; CVOTs, cardiovascular outcomes trials, ACM, all-cause mortality; SAE, serious adverse events; PICO, Population, Intervention, Comparator, Outcome

Domain	Inclusion	Exclusion
Population	Adults (age ≥18 years) with an established diagnosis of HF (any LVEF phenotype: HFrEF ≤40%, HFmrEF 41%-49%, or HFpEF ≥50%)	Type 1 diabetes mellitus as the primary indication; pediatric populations; and non-HF populations
Intervention	GLP-1 RA: semaglutide, liraglutide, tirzepatide, dulaglutide, exenatide, lixisenatide, or albiglutide	Other anti-diabetic agents without GLP-1 RA exposure; non-pharmacologic interventions
Comparator	Placebo or usual care	Active comparator without a placebo arm
Outcomes	At least one outcome of interest reported (CV death, HF hospitalization, ACM, MACE, KCCQ-CSS, 6MWD, body weight, LVEF change, SAEs, and treatment discontinuation due to AEs)	Studies reporting only safety surrogates without efficacy outcomes
Study design	RCT; pre-specified or post-hoc HF subgroup analyses of CVOTs with separately reported HF-specific data	Observational studies; single-arm trials; case reports/series; conference abstracts without full publications; and animal or in vitro studies
Sample size	≥50 participants	<50 participants
Follow-up	≥12 weeks	<12 weeks
Language and date	No language or date restrictions	-

Information Sources and Search Strategy

We searched PubMed (including MEDLINE), Cochrane CENTRAL, and ClinicalTrials.gov from inception through February 3, 2026, without language restrictions. The search strategy combined Medical Subject Headings (MeSH) and free-text terms for GLP-1 RAs (including all individual agent names), HF (including phenotype-specific terms), and RCTs. Embase and Web of Science were not searched because institutional access was unavailable; this database limitation is discussed under the Interpretation section. The full electronic search strategies for each database, including record counts and execution dates, are presented in Table 2.

**Table 2:** Search strategies by database Database-specific Boolean search strings, record counts retrieved, and execution dates. PubMed, Cochrane CENTRAL, and ClinicalTrials.gov were searched without language restrictions through February 3, 2026 (Appendix A). MeSH, Medical Subject Headings

Database	Search strategy	Records retrieved	Date executed
PubMed (including MEDLINE)	("glucagon-like peptide-1"[MeSH] OR "glucagon-like peptide 1"[tiab] OR GLP-1[tiab] OR GLP1[tiab] OR liraglutide[tiab] OR semaglutide[tiab] OR dulaglutide[tiab] OR exenatide[tiab] OR tirzepatide[tiab] OR lixisenatide[tiab] OR albiglutide[tiab] OR "GLP-1 receptor agonist*"[tiab] OR GLP-1RA[tiab] OR "incretin mimetic*"[tiab]) AND ("heart failure"[MeSH] OR "heart failure"[tiab] OR "cardiac failure"[tiab] OR HFrEF[tiab] OR HFpEF[tiab] OR HFmrEF[tiab] OR "preserved ejection fraction"[tiab] OR "reduced ejection fraction"[tiab] OR "systolic dysfunction"[tiab] OR "diastolic dysfunction"[tiab] OR "ventricular dysfunction"[tiab]) AND ("randomized controlled trial"[pt] OR "controlled clinical trial"[pt] OR randomized[tiab] OR randomised[tiab] OR placebo[tiab] OR trial[tiab])	697	2026-02-03
Cochrane CENTRAL	#1 [mh "Glucagon-Like Peptide 1"]; #2 (GLP-1 OR GLP1 OR semaglutide OR liraglutide OR tirzepatide OR dulaglutide OR exenatide):ti,ab,kw; #3 #1 OR #2; #4 [mh "Heart Failure"]; #5 ("heart failure" OR HFrEF OR HFpEF OR HFmrEF):ti,ab,kw; #6 #4 OR #5; #7 #3 AND #6	356	2026-02-03
ClinicalTrials.gov	Condition: Heart Failure | Intervention: GLP-1 OR semaglutide OR liraglutide OR tirzepatide OR dulaglutide OR exenatide | Study Type: Interventional (Clinical Trial)	34	2026-02-03
Total	-	1087	-

Study Selection

Records were imported, combined, and deduplicated using a multi-pass approach (exact Digital Object Identifier (DOI)/PubMed Identifier (PMID) matching, normalized title matching, and fuzzy matching with manual adjudication). Initial screening used algorithmic filters (Python pipeline) to exclude clearly irrelevant records (animal studies, non-RCTs, and non-HF populations). The 200 records that passed the initial filters were then assessed against the full Population, Intervention, Comparator, Outcome (PICO) eligibility framework, with second-reviewer verification of eligibility decisions and all included studies (Appendix B). Full-text eligibility was assessed for all remaining records. Because the initial exclusion stage was not performed as a blinded, parallel dual-reviewer screening process, no inter-rater agreement statistic was calculated; this transparency-first design is acknowledged as a limitation in the Interpretation section. Because the initial searches deviated from the registered strategy, the search was re-executed using the full protocol-compliant strategies, and a screening reconstruction was performed to ensure completeness.

Data Extraction

Data were extracted using a standardized form including: study characteristics (design, agent, dose, population, sample size, and follow-up duration), participant demographics (age, sex, body mass index (BMI), LVEF, and diabetes status), and outcomes (hazard ratios (HRs) with 95% confidence intervals (CIs) for time-to-event outcomes; mean differences (MDs) with standard deviations or 95% CIs for continuous outcomes; and event counts for safety outcomes). For studies reporting only HRs and CIs (without event counts), we computed log(HR) and standard errors using the generic inverse-variance method.

Risk of Bias Assessment

Risk of bias (RoB) was assessed using the Cochrane RoB 2 tool across five domains: randomization process, deviations from intended interventions, missing outcome data, measurement of the outcome, and selection of the reported result [16]. For CVOT subgroup analyses, an additional consideration was applied to the D5 (selective reporting) domain, as HF outcomes were typically secondary or exploratory endpoints.

Effect Measures and Statistical Analysis

The primary outcome was the composite of CV death and first HF hospitalization. Secondary outcomes included all-cause mortality (ACM); major adverse cardiovascular events (MACE; composite of CV death, non-fatal myocardial infarction, and non-fatal stroke); HF hospitalization; CV death; Kansas City Cardiomyopathy Questionnaire Clinical Summary Score (KCCQ-CSS); 6-minute walk distance (6MWD); body weight change; and LVEF change. Safety outcomes included serious adverse events (SAEs) and treatment discontinuation due to adverse events.

For time-to-event outcomes, we used the generic inverse-variance method with log-transformed HRs and corresponding standard errors. Continuous outcomes were pooled as MDs. Safety outcomes (count data) were pooled using the Mantel-Haenszel method with risk ratios (RRs).

Random-effects meta-analysis was performed using restricted maximum likelihood (REML) estimation for the between-study variance (τ²), with the Hartung-Knapp-Sidik-Jonkman (HKSJ) adjustment for CIs [17,18]. Statistical heterogeneity was assessed using I², τ², and Cochran's Q test, with 95% prediction intervals (PIs) to characterize the expected range of effects in future settings [19]. A prespecified subgroup analysis by study type (dedicated HF trial vs. CVOT subgroup) and a dedicated-trials-only sensitivity analysis were used to formally test whether pooling direct and indirect evidence biased the pooled estimate.

Subgroup, Sensitivity, and Publication-Bias Analyses

Prespecified subgroup analyses examined HF phenotype (HFpEF vs. HFrEF/mixed), diabetes status, GLP-1 RA agent, and study type (dedicated HF trial vs. CVOT subgroup); interaction P values were derived using a meta-regression approach. Sensitivity analyses included: (1) fixed-effect (common-effect) meta-analysis; (2) exclusion of the FIGHT trial (patients recently hospitalized for HFrEF); (3) exclusion of tirzepatide (a dual glucose-dependent insulinotropic polypeptide (GIP)/GLP-1 receptor agonist); (4) restriction to dedicated HF trials only; and (5) LOO analysis. Two EXSCEL subgroup analyses (Neves et al. by LVEF; Fudim et al. by HF status) were derived from the same parent trial; the primary analysis used Neves et al. (LVEF-based, closer to the PICO framework), with sensitivity analyses substituting Fudim et al. or excluding both. Publication bias was assessed visually using funnel plots and statistically using Egger's regression test; the trim-and-fill method was applied to estimate the impact of potentially missing studies [20,21]. Trial Sequential Analysis (TSA) was performed for outcomes with available event counts.

Certainty of Evidence

The certainty of evidence was assessed using the GRADE framework across five domains: RoB, inconsistency, indirectness, imprecision, and publication bias [22]. All analyses were performed in R (version 4.4) using the meta and metafor packages. Statistical significance was defined as P<0.05 (two-sided).

Findings

Study Selection

**Figure 1:**
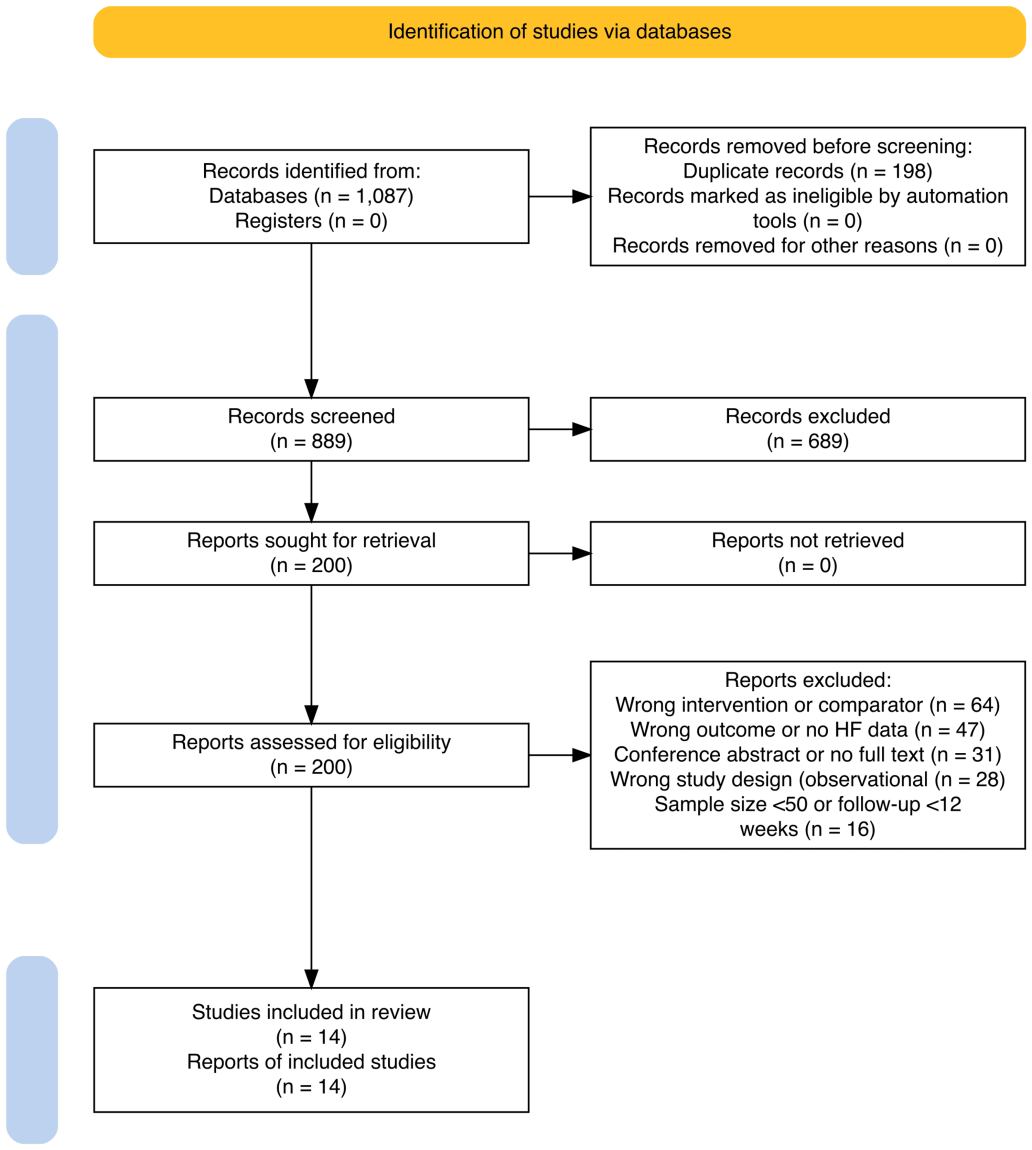
PRISMA 2020 flow diagram of study selection A total of 1087 records were identified across PubMed (n=697), Cochrane CENTRAL (n=356), and ClinicalTrials.gov (n=34). After removing 198 duplicates, 889 unique records underwent title and abstract screening. 200 reports were sought for full-text assessment, 186 were excluded with reasons, and 14 studies were included in quantitative synthesis. HF, heart failure; PRISMA, Preferred Reporting Items for Systematic Reviews and Meta-Analyses

The systematic search identified 1087 records across three databases: PubMed (n=697), Cochrane CENTRAL (n=356), and ClinicalTrials.gov (n=34). After removing 198 duplicates, 889 unique records underwent title and abstract screening. Of these, 689 were excluded, leaving 200 records for full-text assessment. After a detailed eligibility evaluation, 186 records were excluded, and 14 studies met all inclusion criteria and were included in the quantitative synthesis (Figure 1).

Risk of Bias

**Figure 2:**
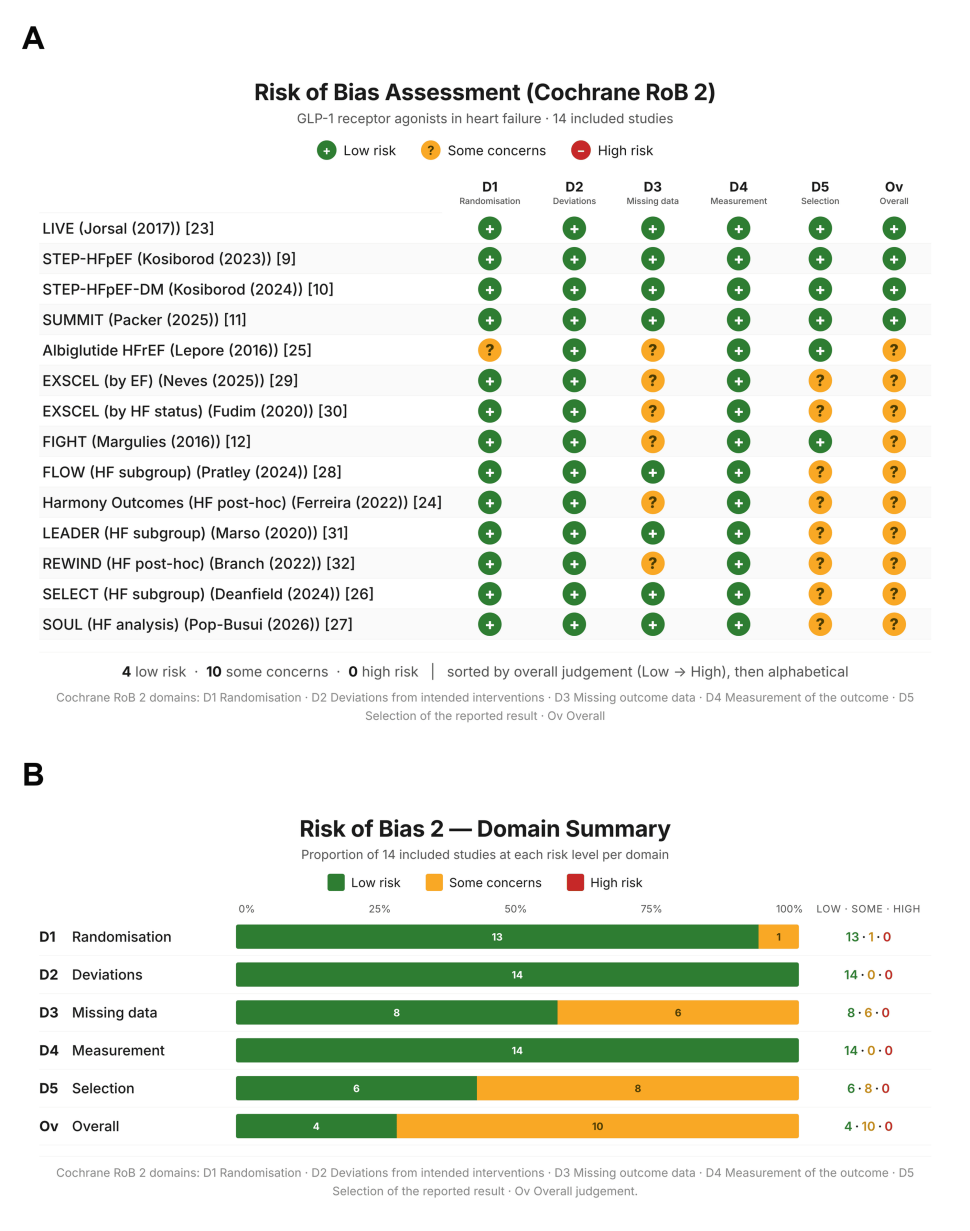
RoB 2 assessment of included studies (composite) Panel A - RoB 2 traffic-light plot by study and domain. Panel B - RoB 2 domain summary across all 14 included studies. Studies displayed (left to right, top to bottom in Panel A): STEP-HFpEF [9], STEP-HFpEF-DM [10], SUMMIT [11], FIGHT [12], Harmony Outcomes (HF post hoc) [24], LIVE [23], Albiglutide HFrEF [25], SELECT (HF subgroup) [26], SOUL (HF analysis) [27], FLOW (HF subgroup) [28], EXSCEL (by EF) [29], EXSCEL (by HF status) [30], LEADER (HF subgroup) [31], and REWIND (HF post hoc) [32]. Color coding: green (low risk), amber (some concerns), red (high risk), and grey (not applicable). RoB, risk of bias; HFpEF, heart failure with preserved ejection fraction; HF, heart failure

Four studies were judged as low RoB (SUMMIT [11], STEP-HFpEF [9], STEP-HFpEF-DM [10], LIVE [23]), and 10 studies had some concerns, primarily related to post-hoc or subgroup analysis design affecting the selective reporting domain (D5). No studies were rated as high RoB (Figure 2).

Study Characteristics

The 14 included studies encompassed 18,184 patients in HF subgroups or dedicated HF arms, of whom 2499 were directly randomized in six dedicated HF trials (SUMMIT [11], STEP-HFpEF [9], STEP-HFpEF-DM [10], FIGHT [12], LIVE [23], and Albiglutide HFrEF [25]), with the remainder enrolled through eight CVOT HF subgroup analyses (Harmony Outcomes HF [24], SELECT HF [26], SOUL HF [27], FLOW HF [28], EXSCEL by ejection fraction (EF) [29], LEADER HF [31], REWIND HF [32], and EXSCEL by HF status [30]). For the primary composite, only two studies (SUMMIT [11] and FIGHT [12]; 1031 patients) provided adjudicated event counts; the remaining six studies contributing to the primary composite provided HRs without individual event data. The GLP-1 RAs studied included semaglutide (5 studies), liraglutide (3 studies), exenatide (2 studies), albiglutide (2 studies), dulaglutide (1 study), and tirzepatide (1 study). Mean participant age ranged from 61 to 69 years, female participation ranged from 15% to 56%, and follow-up ranged from 12 to 273 weeks. Trial-level characteristics are summarized in Table 3, and per-study RoB 2 domain judgments are detailed in Table 4.

**Table 3:** Characteristics of included studies Trial-level characteristics of the 14 included studies, with reference numbers in the Ref column. Each row reports one of the included studies. Tx, treatment; Ctrl, control; FU, follow-up (weeks); RoB 2, risk of bias 2; HFrEF, heart failure with reduced ejection fraction; HFpEF, heart failure with preserved ejection fraction; CVOT, cardiovascular outcomes trial; GIP, glucose-dependent insulinotropic polypeptide; GLP-1RA, glucagon-like peptide-1 receptor agonist; HF, heart failure; BMI, body mass index; EF, ejection fraction; DM, diabetes mellitus

Ref	Study	Trial	Agent	Agent class	Study type	HF phenotype	N (Tx)	N (Ctrl)	Mean Age	Female (%)	Mean BMI	Mean LVEF (%)	FU (wk)	Diabetes (%)	DM status	RoB 2
[9]	Kosiborod (2023)	STEP-HFpEF	Semaglutide	GLP-1 RA	Dedicated HF trial	HFpEF	263	266	69	56	37	57	52	0	No DM	Low
[10]	Kosiborod (2024)	STEP-HFpEF-DM	Semaglutide	GLP-1 RA	Dedicated HF trial	HFpEF	310	306	69	44	37	57	52	100	All DM	Low
[11]	Packer (2025)	SUMMIT	Tirzepatide	Dual GIP/GLP-1	Dedicated HF trial	HFpEF	364	367	65.2	53.8	38.4	61	104	62	Mixed	Low
[12]	Margulies (2016)	FIGHT	Liraglutide	GLP-1 RA	Dedicated HF trial	HFrEF	154	146	61	21	33	25	24	57	Mixed	Some concerns
[23]	Jorsal (2017)	LIVE	Liraglutide	GLP-1 RA	Dedicated HF trial	HFrEF	122	119	64	15	30	34	24	36	Mixed	Low
[24]	Ferreira (2022)	Harmony Outcomes (HF post-hoc)	Albiglutide	GLP-1 RA	CVOT HF subgroup	Mixed	505	524	65	24	33	NR	83	100	All DM	Some concerns
[25]	Lepore (2016)	Albiglutide HFrEF	Albiglutide	GLP-1 RA	Dedicated HF trial	HFrEF	55	27	61	15	31	30	12	35	Mixed	Some concerns
[26]	Deanfield (2024)	SELECT (HF subgroup)	Semaglutide	GLP-1 RA	CVOT HF subgroup	Mixed	2145	2141	63	28	33.4	NR	175	72	Mixed	Some concerns
[27]	Pop-Busui (2026)	SOUL (HF analysis)	Semaglutide	GLP-1 RA	CVOT HF subgroup	Mixed	1104	1125	66	29	NA	NR	206	100	All DM	Some concerns
[28]	Pratley (2024)	FLOW (HF subgroup)	Semaglutide	GLP-1 RA	CVOT HF subgroup	Mixed	811	810	67	30	32.5	NR	167	100	All DM	Some concerns
[29]	Neves (2025)	EXSCEL (by EF)	Exenatide	GLP-1 RA	CVOT HF subgroup	Mixed	808	824	63	33	33	NR	167	100	All DM	Some concerns
[30]	Fudim (2020)	EXSCEL (by HF status)	Exenatide	GLP-1 RA	CVOT HF subgroup	Mixed	960	981	63	32	33	NR	167	100	All DM	Some concerns
[31]	Marso (2020)	LEADER (HF subgroup)	Liraglutide	GLP-1 RA	CVOT HF subgroup	Mixed	835	832	64	19	33	NR	197	100	All DM	Some concerns
[32]	Branch (2022)	REWIND (HF post-hoc)	Dulaglutide	GLP-1 RA	CVOT HF subgroup	Mixed	631	649	67	37	33	NR	273	100	All DM	Some concerns

**Table 4:** RoB 2: per-study domain judgments Cochrane RoB 2 (RoB 2) per-study judgments across the five core domains plus an overall judgment. Each row reports one of the 14 included studies, with reference numbers in the Ref column and a final column indicating post hoc subgroup status for CVOT analyses. D1, randomization process; D2, deviations from intended interventions; D3, missing outcome data; D4, measurement of the outcome; D5, selection of the reported result. CVOT, cardiovascular outcomes trial; RoB: risk of bias; HFpEF, heart failure with preserved ejection fraction; HF, heart failure; HFrEF, heart failure with reduced ejection fraction; DM, diabetes mellitus; EF, ejection fraction

Ref	Study	Trial	D1: randomisation	D2: deviations	D3: missing data	D4: measurement	D5: selection	Overall	Post-hoc subgroup
[9]	Kosiborod (2023)	STEP-HFpEF	Low	Low	Low	Low	Low	Low	No
[10]	Kosiborod (2024)	STEP-HFpEF-DM	Low	Low	Low	Low	Low	Low	No
[11]	Packer (2025)	SUMMIT	Low	Low	Low	Low	Low	Low	No
[12]	Margulies (2016)	FIGHT	Low	Low	Some concerns	Low	Low	Some concerns	No
[23]	Jorsal (2017)	LIVE	Low	Low	Low	Low	Low	Low	No
[24]	Ferreira (2022)	Harmony Outcomes (HF post-hoc)	Low	Low	Some concerns	Low	Some concerns	Some concerns	Yes, post-hoc HF subgroup of CVOT
[25]	Lepore (2016)	Albiglutide HFrEF	Some concerns	Low	Some concerns	Low	Low	Some concerns	No
[26]	Deanfield (2024)	SELECT (HF subgroup)	Low	Low	Low	Low	Some concerns	Some concerns	Yes, prespecified subgroup of CVOT
[27]	Pop-Busui (2026)	SOUL (HF analysis)	Low	Low	Low	Low	Some concerns	Some concerns	Yes, secondary analysis of parent CVOT
[28]	Pratley (2024)	FLOW (HF subgroup)	Low	Low	Low	Low	Some concerns	Some concerns	Yes, post-hoc HF subgroup of renal trial
[29]	Neves (2025)	EXSCEL (by EF)	Low	Low	Some concerns	Low	Some concerns	Some concerns	Yes, post-hoc EF-stratified analysis
[30]	Fudim (2020)	EXSCEL (by HF status)	Low	Low	Some concerns	Low	Some concerns	Some concerns	Yes, post-hoc HF subgroup of CVOT
[31]	Marso (2020)	LEADER (HF subgroup)	Low	Low	Low	Low	Some concerns	Some concerns	Yes, prespecified subgroup but HF exploratory
[32]	Branch (2022)	REWIND (HF post-hoc)	Low	Low	Some concerns	Low	Some concerns	Some concerns	Yes, post-hoc HF subgroup of CVOT

Primary Outcome: Composite of Cardiovascular Death and Heart Failure Hospitalization

**Figure 3:**
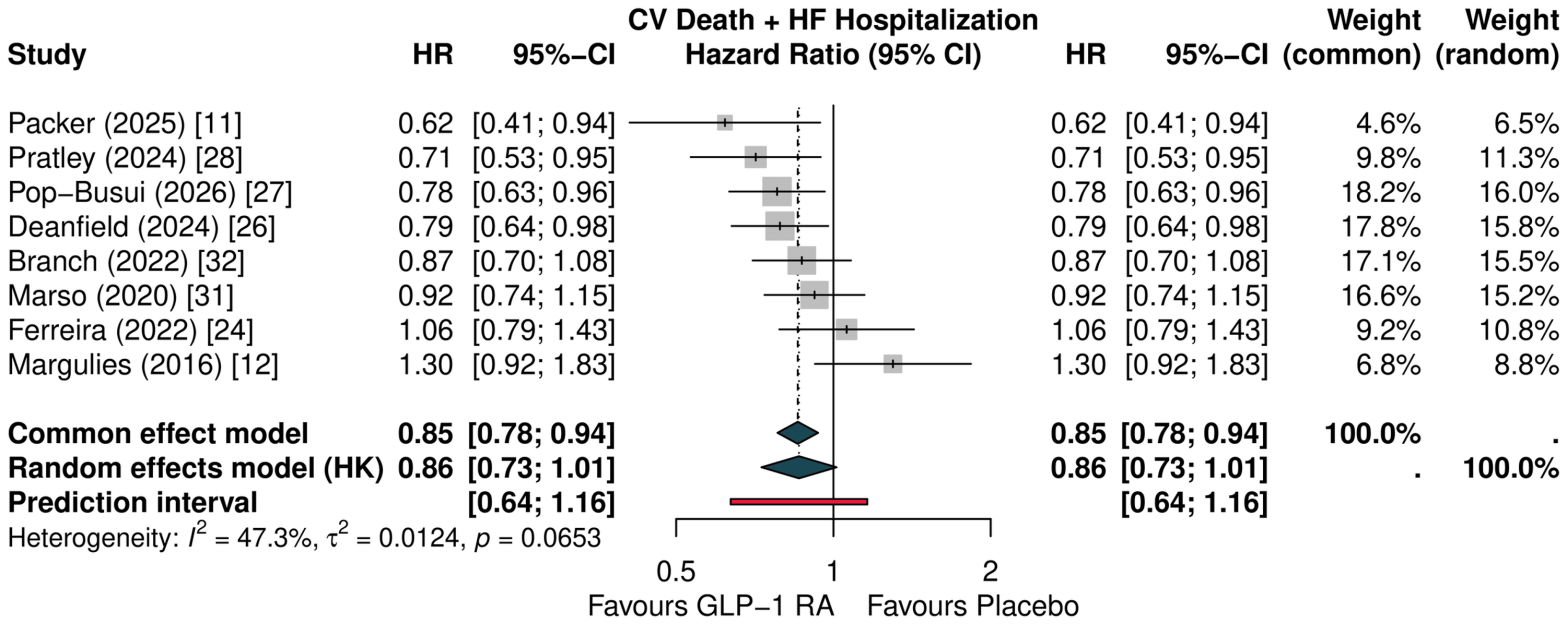
Forest plot of the primary composite outcome (CV death and first HF hospitalization) HRs with 95% CIs from a random-effects meta-analysis (REML + HKSJ). Studies displayed (top to bottom): SUMMIT [11], FIGHT [12], SELECT (HF subgroup) [26], SOUL (HF analysis) [27], FLOW (HF subgroup) [28], Harmony Outcomes (HF post hoc) [24], LEADER (HF subgroup) [31], and REWIND (HF post hoc) [32]. The diamond represents the pooled estimate. CI, confidence interval; CV, cardiovascular; HF, heart failure; HKSJ, Hartung-Knapp-Sidik-Jonkman; HR, hazard ratio; REML, restricted maximum likelihood; GLP-1RA, glucagon-like peptide-1 receptor agonist

Eight studies contributed data for the primary composite outcome: SUMMIT [11], FIGHT [12], SELECT HF [26], SOUL HF [27], FLOW HF [28], Harmony Outcomes HF [24], LEADER HF [31], and REWIND HF [32]. The primary composite did not reach statistical significance, and effect estimates spanned both benefit and harm. The pooled HR was 0.86 (95% CI 0.73-1.01; P=0.067) using the HKSJ random-effects model, with moderate heterogeneity (I²=47%, τ²=0.022). The 95% PI ranged from 0.64 to 1.16, suggesting that future studies may show effects ranging from substantial benefit to modest harm. Individual study estimates ranged from HR 0.62 (SUMMIT [11]) to HR 1.30 (FIGHT [12]), with most studies favoring GLP-1 RAs (Figure 3). Pooled estimates for all primary and secondary outcomes are summarized in Table 5.

**Table 5:** Summary of meta-analysis results Pooled estimates by outcome, including the number of studies (k), HRs, RRs, or mean differences, 95% CIs, P values, I², and certainty of evidence according to GRADE. ACM, all-cause mortality; MACE, major adverse cardiovascular events; HF, heart failure; CV, cardiovascular; KCCQ-CSS, Kansas City Cardiomyopathy Questionnaire Clinical Summary Score; 6MWD, 6-minute walk distance; LVEF, left ventricular ejection fraction; HR, hazard ratio; RR, risk ratio; MD, mean difference; CI, confidence interval; GRADE, Grading of Recommendations, Assessment, Development, and Evaluations PI, prediction interval; HKSJ, Hartung-Knapp-Sidik-Jonkman

Outcome	Measure	k	Estimate	95% CI	P (HKSJ)	I² (%)	PI
CV death+HF hospitalization (primary)	HR	8	0.86	(0.73, 1.01)	0.0672	47.3	(0.64, 1.16)
HF hospitalization	HR	5	0.87	(0.62, 1.22)	0.3177	59.3	(0.45, 1.67)
ACM	HR	7	0.86	(0.79, 0.95)	0.0085	0.0	(0.75, 1.00)
CV death	HR	3	0.79	(0.53, 1.19)	0.1348	0.0	(0.50, 1.25)
MACE	HR	4	0.80	(0.69, 0.93)	0.0168	0.0	(0.68, 0.95)
KCCQ-CSS	MD (pts)	3	7.4	(6.3, 8.5)	0.0012	0.0	(3.3, 11.5)
6MWD	MD (m)	4	17.6	(13.4, 21.7)	0.0009	0.0	(8.4, 26.7)
Weight change	MD (kg)	3	-9.3	(-15.8, -2.7)	0.0261	94.4	(-22.1, 3.6)
LVEF change	MD (%)	2	0.8	(-24.5, 26.0)	0.7695	72.5	-

All-Cause Mortality

**Figure 4:**
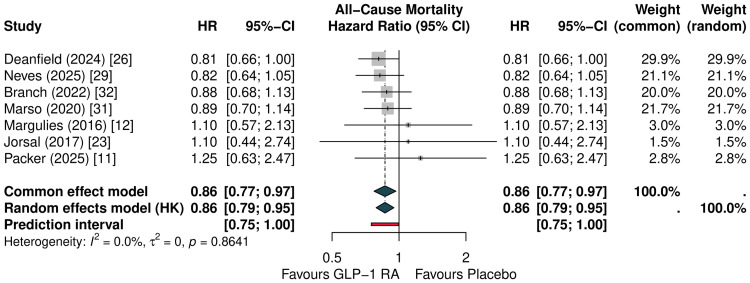
Forest plot of ACM Pooled HR 0.86 (95% CI 0.79 to 0.95; I²=0%) across seven studies. Studies displayed (top to bottom): SUMMIT [11], FIGHT [12], LIVE [23], SELECT (HF subgroup) [26], SOUL (HF analysis) [27], LEADER (HF subgroup) [31], and EXSCEL (by EF) [29]. ACM, all-cause mortality; CI, confidence interval; HR, hazard ratio; GLP-1RA, glucagon-like peptide-1 receptor agonist

GLP-1 RAs significantly reduced ACM across seven studies: SUMMIT [11], FIGHT [12], LIVE [23], SELECT HF [26], SOUL HF [27], LEADER HF [31], and EXSCEL by EF [29] (HR 0.86, 95% CI 0.79-0.95; P=0.008; I²=0%), with a PI of 0.75 to 1.00. Because the upper bound of the PI approaches the null, this finding should not be interpreted as evidence that the mortality benefit would be consistent across all future HF populations. Notably, this pooled benefit was driven by CVOT subgroups; among dedicated HF trials, SUMMIT [11] showed numerically higher CV death (HR 1.58; 8 vs. 5 events) and ACM (HR 1.25; 19 vs. 15 deaths) with tirzepatide, and both FIGHT [12] (HR 1.10) and LIVE [23] (HR 1.10) showed a similar direction of effect. The GRADE certainty was rated as low (downgraded for indirectness due to reliance on CVOT subgroups and for incoherence given the divergent signals between indirect and direct evidence). Sensitivity analyses substituting Fudim [30] for Neves [29] yielded an HR of 0.88 (95% CI 0.81-0.95), and excluding both EXSCEL subgroups yielded an HR of 0.88 (95% CI 0.78-0.98), demonstrating directional consistency (Figure 4).

Major Adverse Cardiovascular Events

**Figure 5:**
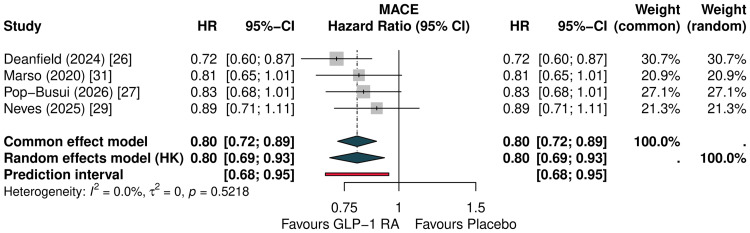
Forest plot of MACE Pooled HR 0.80 (95% CI 0.69 to 0.93; I²=0%) across 4 studies. Studies displayed (top to bottom): SELECT (HF subgroup) [26], FLOW (HF subgroup) [28], LEADER (HF subgroup) [31], EXSCEL (by EF) [29]. MACE, major adverse cardiovascular events; CI, confidence interval; HR, hazard ratio

MACE was significantly reduced in the four studies reporting this outcome: SELECT HF [26], FLOW HF [28], LEADER HF [31], and Neves EXSCEL (by EF) [29] (after exclusion of the overlapping EXSCEL by HF status subgroup [30]), yielding an HR of 0.80 (95% CI 0.69-0.93; P=0.017; I²=0%), with consistent effects and moderate-certainty GRADE evidence (Figure 5).

Heart Failure Hospitalization

**Figure 6:**
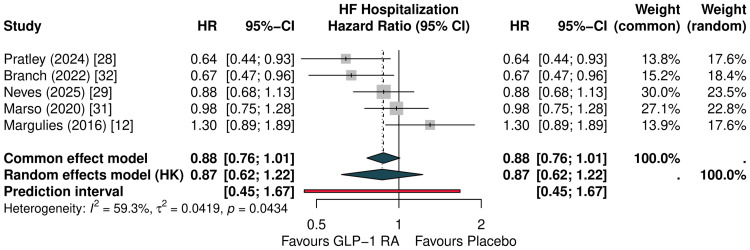
Forest plot of HF hospitalization Pooled HR 0.87 (95% CI 0.62 to 1.22; I²=59%) across five studies. Studies displayed (top to bottom): SUMMIT [11], FIGHT [12], FLOW (HF subgroup) [28], Harmony Outcomes (HF post-hoc) [24], EXSCEL (by EF) [29]. HF, heart failure; CI, confidence interval; HR, hazard ratio; GLP-1RA, glucagon-like peptide-1 receptor agonist; HF, heart failure; EF, ejection fraction

HF hospitalization showed a non-significant trend toward reduction across five studies: SUMMIT [11], FIGHT [12], FLOW HF [28], Harmony Outcomes HF [24], and Neves EXSCEL (by EF) [29] (after excluding the overlapping EXSCEL by HF status subgroup [30]), yielding an HR of 0.87 (95% CI 0.62-1.22; P=0.318; I²=59%), with moderate heterogeneity driven by divergent results between dedicated HF trials and CVOT subgroups (Figure 6).

*Cardiovascular* *Death*

**Figure 7:**
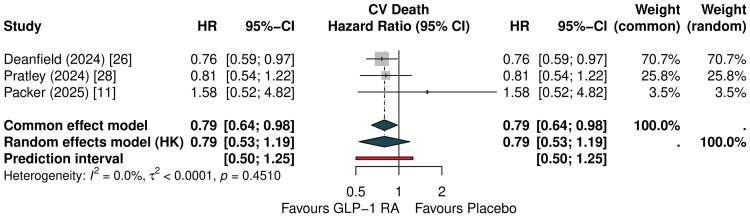
Forest plot of CV death HRs with 95% CIs from random-effects meta-analysis (REML+HKSJ). Pooled HR 0.79 (95% CI 0.53 to 1.19; I²=0%) across three studies. Studies displayed (top to bottom): SUMMIT [11], SELECT (HF subgroup) [26], SOUL (HF analysis) [27]. CV, cardiovascular; CI, confidence interval; HR, hazard ratio; HKSJ, Hartung-Knapp-Sidik-Jonkman; REML, restricted maximum likelihood; GLP-1RA, glucagon-like peptide-1 receptor agonist; HF, heart failure

CV death showed a non-significant reduction (HR 0.79, 95% CI 0.53-1.19; P=0.135; I²=0%; k=3) across SUMMIT [11], SELECT HF [26], and SOUL HF [27], although this analysis was limited by the small number of contributing studies (Figure 7).

Functional and Patient-Reported Outcomes

**Figure 8:**
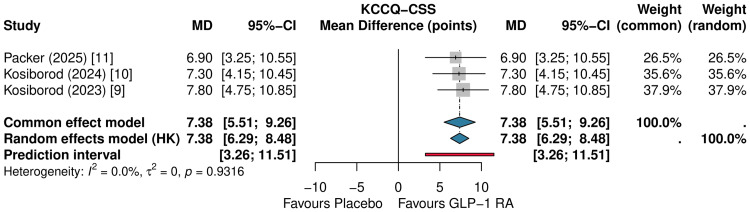
Forest plot of KCCQ-CSS Pooled mean difference +7.4 points (95% CI 6.3 to 8.5; I²=0%) across three studies. Studies displayed (top to bottom): SUMMIT [11], STEP-HFpEF [9], STEP-HFpEF-DM [10]. KCCQ-CSS, Kansas City Cardiomyopathy Questionnaire Clinical Summary Score; CI, confidence interval; MD, mean difference; GLP-1RA, glucagon-like peptide-1 receptor agonist; HFpEF, heart failure with preserved ejection fraction; DM, diabetes mellitus

**Figure 9:**
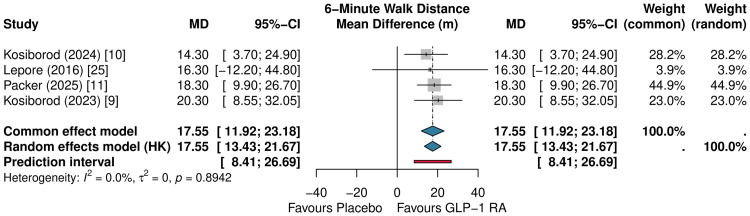
Forest plot of 6MWD Pooled mean difference +17.6 m (95% CI 13.4 to 21.7; I²=0%) across four studies. Studies displayed (top to bottom): SUMMIT [11], STEP-HFpEF [9], STEP-HFpEF-DM [10], Albiglutide HFrEF [25]. 6MWD, 6-minute walk distance; CI, confidence interval; MD, mean difference; GLP-1RA, glucagon-like peptide-1 receptor agonist; HFpEF, heart failure with preserved ejection fraction; DM, diabetes mellitus

GLP-1 RAs produced clinically meaningful improvements in both patient-reported outcomes and functional capacity. KCCQ-CSS improved by a mean of 7.4 points (95% CI 6.3-8.5; P=0.001; I²=0%; k=3) in the dedicated HFpEF trials SUMMIT [11], STEP-HFpEF [9], and STEP-HFpEF-DM [10], exceeding the minimal clinically important difference of five points. The 6MWD improved by 17.6 m (95% CI 13.4-21.7; P<0.001; I²=0%; k=4) across SUMMIT [11], STEP-HFpEF [9], STEP-HFpEF-DM [10], and Albiglutide HFrEF [25], also surpassing clinically meaningful thresholds. The pooled effects for KCCQ-CSS and 6MWD are shown in Figure 8 and Figure 9, respectively.

Body Weight and Left Ventricular Ejection Fraction

**Figure 10:**
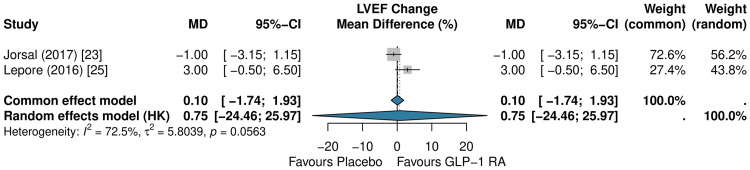
Forest plot of LVEF change Mean differences with 95% CIs from random-effects meta-analysis (REML+HKSJ). Pooled MD 0.8% (95% CI: -24.5 to 26.0; I²=73%) across two studies. Studies displayed (top to bottom): LIVE [23], Albiglutide HFrEF [25]. LVEF, left ventricular ejection fraction; CI, confidence interval; MD, mean difference; HKSJ, Hartung-Knapp-Sidik-Jonkman; REML, restricted maximum likelihood; GLP-1RA, glucagon-like peptide-1 receptor agonist; HFrEF, heart failure with reduced ejection fraction

**Figure 11:**
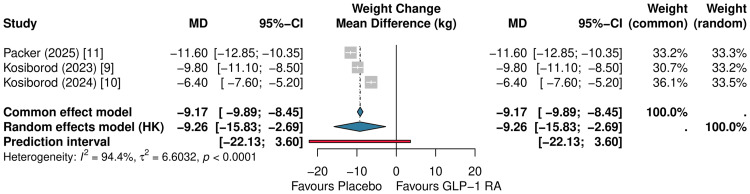
Forest plot of body weight change Mean differences with 95% CIs from random-effects meta-analysis (REML+HKSJ). Pooled MD −9.3 kg (95% CI -15.8 to -2.7; I²=94%) across three studies. Studies displayed (top to bottom): SUMMIT [11], STEP-HFpEF [9], STEP-HFpEF-DM [10]. CI, confidence interval; MD, mean difference; HKSJ, Hartung-Knapp-Sidik-Jonkman; REML, restricted maximum likelihood; HFpEF, heart failure with preserved ejection; fraction; GLP-1RA, glucagon-like peptide-1 receptor agonist; DM, diabetes mellitus

Weight reduction was substantial (MD -9.3 kg, 95% CI -15.8 to -2.7; P=0.026; I²=94%; k=3), although heterogeneity was high, reflecting differences in agents, doses, and study populations. LVEF change was not significantly different between groups (MD 0.8%, 95% CI -24.5 to 26.0; P=0.770; I²=73%; k=2). The pooled effects for LVEF and body weight are shown in Figure 10 and Figure 11, respectively.

Safety

**Figure 12:**
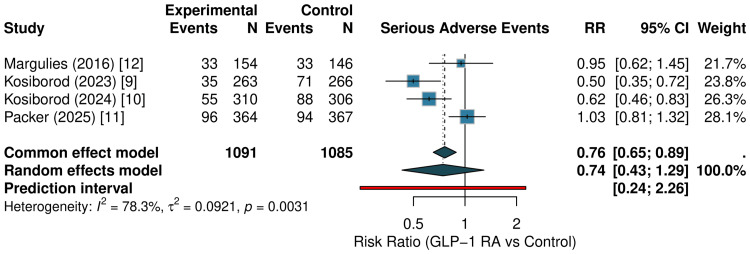
Forest plot of SAE RRs from Mantel-Haenszel random-effects meta-analysis. Pooled RR 0.74 (95% CI 0.43 to 1.29; I²=78%) across four studies. Studies displayed (top to bottom): SUMMIT [11], STEP-HFpEF [9], STEP-HFpEF-DM [10], FIGHT [12]. SAE, serious adverse event; CI, confidence interval; RR, risk ratio; GLP-1RA, glucagon-like peptide-1 receptor agonist; HFpEF, heart failure with preserved ejection fraction; DM, diabetes mellitus

**Figure 13:**
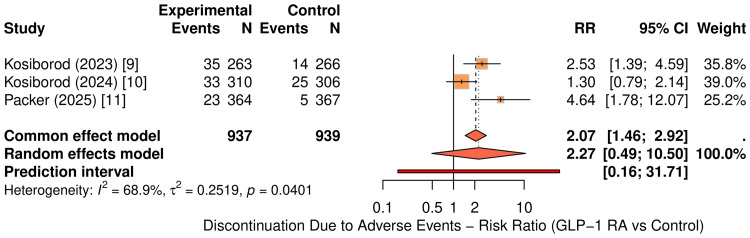
Forest plot of treatment discontinuation due to adverse events RRs from Mantel-Haenszel random-effects meta-analysis. Pooled RR 2.27 (95% CI 0.49 to 10.50; I²=69%) across three studies. Studies displayed (top to bottom): SUMMIT [11], STEP-HFpEF [9], STEP-HFpEF-DM [10]. AE, adverse event; CI, confidence interval; RR, risk ratio; GLP-1RA, glucagon-like peptide-1 receptor agonist; HFpEF, heart failure with preserved ejection fraction; DM, diabetes mellitus

Safety analyses were restricted to dedicated HF trials with available per-arm data. SAEs showed a non-significant trend toward fewer events with GLP-1 RAs (RR 0.74, 95% CI 0.43-1.29; P=0.186; I²=78%; k=4) across SUMMIT [11], STEP-HFpEF [9], STEP-HFpEF-DM [10], and FIGHT [12], with high heterogeneity reflecting divergent results between HFpEF trials (favoring GLP-1 RAs) and FIGHT in HFrEF (neutral). Treatment discontinuation due to adverse events was numerically higher with GLP-1 RAs (RR 2.27, 95% CI 0.49-10.50; P=0.147; I²=69%; k=3) across SUMMIT [11], STEP-HFpEF [9], and STEP-HFpEF-DM [10], consistent with the known gastrointestinal tolerability profile of this drug class. SAEs are shown in Figure 12, and treatment discontinuation due to adverse events is shown in Figure 13.

Sensitivity Analyses for the Primary Composite

**Figure 14:**
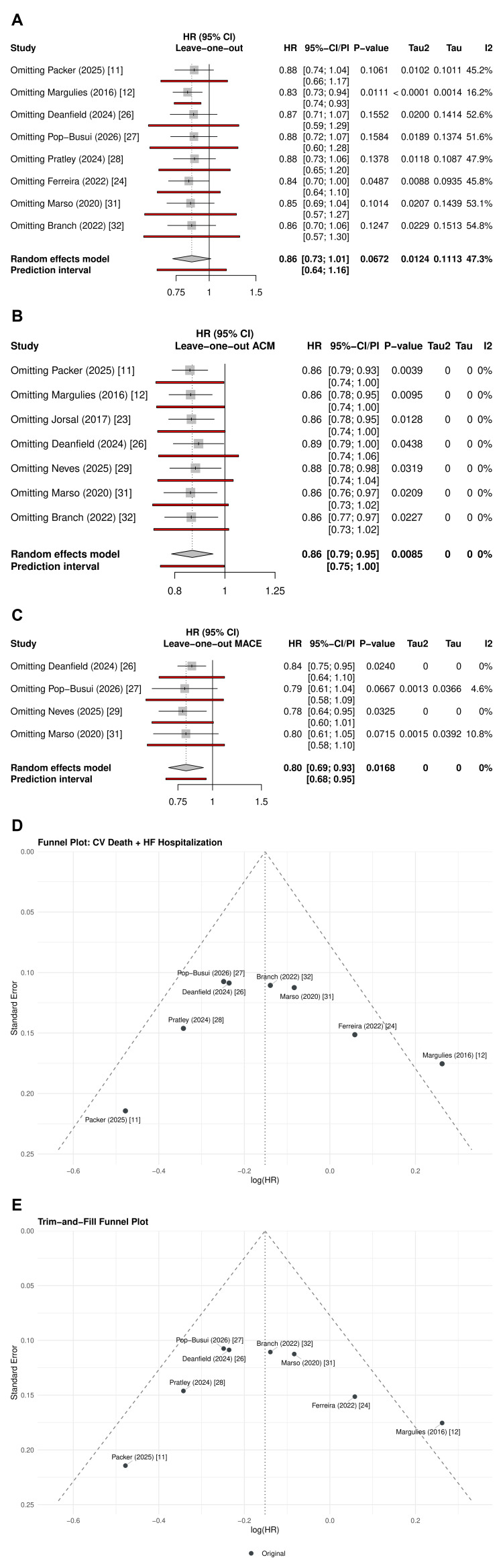
Composite outcome, sensitivity analyses, and publication bias Panel A - LOO plot for the primary composite (k=8: SUMMIT [11], FIGHT [12], SELECT (HF subgroup) [26], SOUL (HF analysis) [27], FLOW (HF subgroup) [28], Harmony Outcomes (HF post hoc) [24], LEADER (HF subgroup) [31], and REWIND (HF post hoc) [32]). Panel B - LOO plot for ACM (k=7: SUMMIT [11], FIGHT [12], LIVE [23], SELECT (HF subgroup) [26], SOUL (HF analysis) [27], LEADER (HF subgroup) [31], and EXSCEL (by EF) [29]). Panel C - LOO plot for MACE (k=4: SELECT (HF subgroup) [26], FLOW (HF subgroup) [28], LEADER (HF subgroup) [31], and EXSCEL (by EF) [29]). Panel D - funnel plot for the primary composite (Egger's test P=0.38; k=8). Panel E - trim-and-fill funnel plot estimating no missing studies. ACM, all-cause mortality; MACE, major adverse cardiovascular events, LOO: leave-one-out; HR, hazard ratio; CI, confidence interval; PI, prediction interval; CV, cardiovascular; HF, heart failure

The fixed-effect (common-effect) model yielded a significant pooled HR of 0.85 (95% CI 0.78-0.94; P=0.0006). The difference between the fixed- and random-effects results reflects the impact of between-study heterogeneity on CI width under the HKSJ method. Excluding the FIGHT trial [12] resulted in a significant pooled HR of 0.83 (95% CI 0.73-0.94; P=0.011) with substantially reduced heterogeneity (I²=16%), confirming FIGHT as a key source of heterogeneity. Excluding tirzepatide (SUMMIT [11]) had minimal impact (HR 0.88, 95% CI 0.74-1.04; P=0.106). Restricting the analysis to dedicated HF trials reporting the primary composite (k=2: SUMMIT [11] and FIGHT [12]) yielded an HR of 0.91 (95% CI 0.01-99.93; P=0.836; I²=86%), with extremely wide CIs reflecting the small number of dedicated trials with primary composite event data and the stark contrast between their results. LOO analysis confirmed that no single study disproportionately influenced the overall result beyond FIGHT [12]. The full set of sensitivity analyses is reported in Table 6 (Appendix C), and the LOO and publication-bias diagnostics are shown in Figure 14.

**Table 6:** Sensitivity analyses for the primary composite outcome Pre-specified and post-hoc sensitivity analyses for the primary composite. Each row reports k studies and a pooled estimate with 95% CI and P value. HR, hazard ratio; CI, confidence interval; CVOT, cardiovascular outcomes trial

Analysis	k	HR	95% CI	P	I² (%)
Primary (all studies)	8	0.86	(0.73, 1.01)	0.0672	47.3
Fixed-effect	8	0.85	(0.78, 0.94)	0.0006	47.3
Excl. Tirzepatide	7	0.88	(0.74, 1.04)	0.1061	45.2
Excl. FIGHT	7	0.83	(0.73, 0.94)	0.0111	16.2
Dedicated HF trials only	2	0.91	(0.01, 99.93)	0.8359	86.0
Excl. CVOT post-hoc subgroups	2	0.91	(0.01, 99.93)	0.8359	86.0

Subgroup Analyses

**Figure 15:**
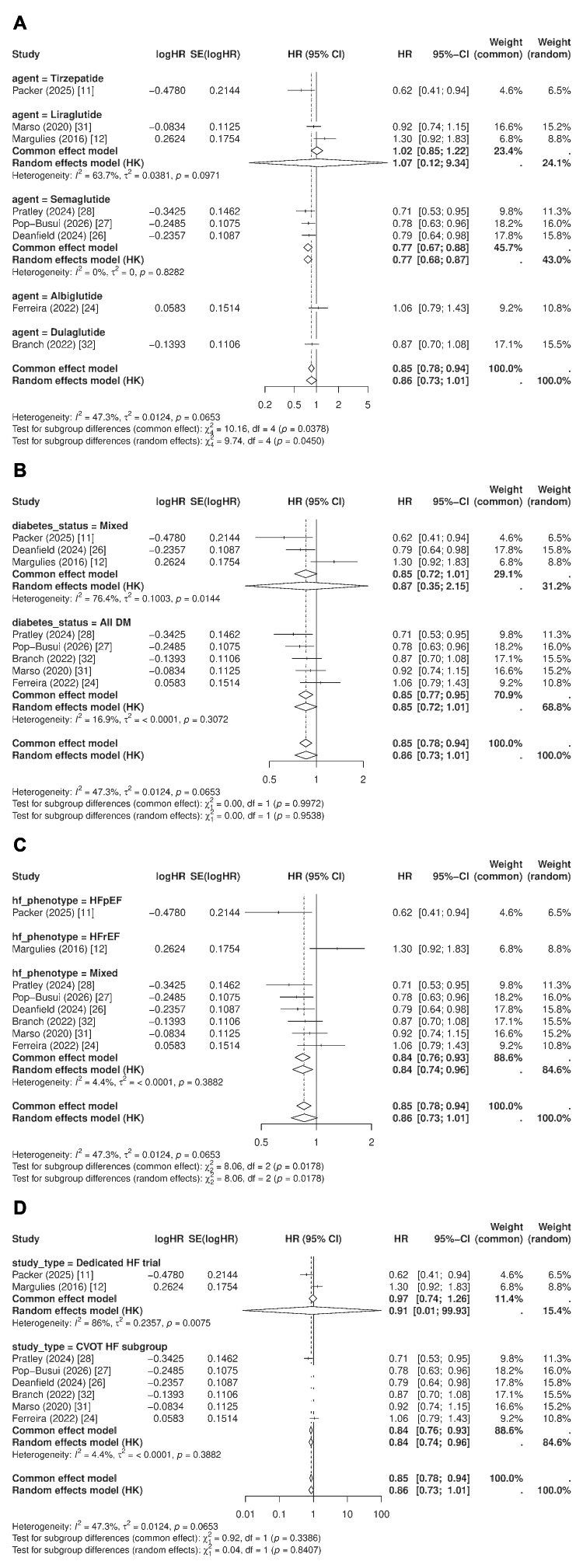
Subgroup analyses of the primary composite outcome Panel A - by GLP-1 RA agent (P-interaction=0.045). Panel B - by diabetes status (P-interaction=0.954). Panel C - by HF phenotype (HFpEF vs. HFrEF/mixed; P-interaction=0.018). Panel D - by study type (dedicated HF trial vs. CVOT subgroup; P-interaction=0.841). Pooled estimates for each subgroup with 95% CIs across the eight studies contributing to the primary composite: SUMMIT [11], FIGHT [12], SELECT (HF subgroup) [26], SOUL (HF analysis) [27], FLOW (HF subgroup) [28], Harmony Outcomes (HF post hoc) [24], LEADER (HF subgroup) [31], and REWIND (HF post hoc) [32]. The HF phenotype and agent interactions are constrained by single-study strata (HFpEF: SUMMIT only; HFrEF: FIGHT only) and should be interpreted cautiously. GLP-1 RA, glucagon-like peptide-1 receptor agonist; HF, heart failure; HFpEF, heart failure with preserved ejection fraction; HFrEF, heart failure with reduced ejection fraction; CVOT, cardiovascular outcomes trial; CI, confidence interval; HR, hazard ratio; DM, diabetes mellitus

Subgroup analyses by HF phenotype showed a significant interaction (P-interaction=0.018): HFpEF-focused studies showed clear benefit (HR 0.62, 95% CI 0.41-0.94; SUMMIT [11]), HFrEF showed a directionally unfavorable effect (HR 1.30, 95% CI 0.92-1.83; FIGHT [12]), and mixed-phenotype CVOT subgroups showed modest benefit (HR 0.84, 95% CI 0.74-0.96; k=6). Subgroup analysis by GLP-1 RA agent also reached significance (P-interaction=0.045), with semaglutide showing the most consistent benefit (HR 0.77, 95% CI 0.68-0.87; k=3). Analyses by diabetes status (P-interaction=0.954) and study type (dedicated HF trial vs. CVOT HF subgroup; P-interaction=0.841) did not reveal significant interactions, likely reflecting limited statistical power within strata. The HF phenotype interaction should be interpreted cautiously, given that the HFpEF and HFrEF strata each contained a single study (SUMMIT and FIGHT, respectively); confounding among phenotype, agent, and trial design cannot be excluded. All eight studies contributing to the primary composite (SUMMIT [11], FIGHT [12], SELECT HF [26], SOUL HF [27], FLOW HF [28], Harmony Outcomes HF [24], LEADER HF [31], and REWIND HF [32]) participated in at least one subgroup comparison (Figure 15).

Trial Sequential Analysis

TSA was performed for the primary composite outcome and ACM using studies with available event counts. For the primary composite outcome, only SUMMIT [11] and FIGHT [12] provided event counts (k=2), and the required information size was not reached under either 15% or 20% relative risk reduction assumptions. For ACM, three dedicated trials provided event counts (SUMMIT [11], FIGHT [12], and LIVE [23]; k=3); TSA similarly did not reach the required information size, indicating that the current evidence is insufficient to draw definitive conclusions regarding the true effect size. TSA was not feasible for MACE because no studies reported event counts for this outcome; all MACE data were derived from CVOT subgroups reporting only HRs and CIs.

Certainty of Evidence (Grading of Recommendations, Assessment, Development, and Evaluations)

Using the GRADE framework, the certainty of evidence was low for ACM (downgraded for indirectness and incoherence, given the divergent signals between CVOT subgroups favoring benefit and dedicated HF trials showing a directionally unfavorable effect), moderate for MACE, low for the primary composite outcome and HF hospitalization (downgraded for indirectness and imprecision), and moderate for KCCQ-CSS and 6MWD. The primary sources of downgrading were indirectness (CVOT subgroup analyses in which HF was not the primary trial endpoint) and, for ACM specifically, incoherence between indirect and direct evidence. The full GRADE evidence profile is presented in Table 7.

**Table 7:** GRADE evidence profile ^1^Outcome derived predominantly from CVOT subgroup analyses in which heart failure was not the primary trial endpoint. ^2^Wide confidence interval and/or limited direct evidence (adjudicated event counts available from only two dedicated heart failure trials). ^3^Divergent signals between CVOT subgroups (favoring benefit) and dedicated heart failure trials (showing directional harm). GRADE evidence profile across the seven primary and secondary outcomes, including judgments for RoB, inconsistency, indirectness, imprecision, publication bias, and the final certainty rating. GRADE, Grading of Recommendations, Assessment, Development, and Evaluations; ACM, all-cause mortality; MACE, major adverse cardiovascular events; HF, heart failure; KCCQ-CSS, Kansas City Cardiomyopathy Questionnaire Clinical Summary Score; 6MWD, 6-minute walk distance; CVOT, cardiovascular outcomes trial; RoB, risk of bias

Outcome	N studies (participants)	RoB	Inconsistency	Indirectness	Imprecision	Publication bias	Certainty
CV death+HF hospitalization	8 (13143)	Not serious	Moderate (I²=47%)	Serious^1^	Serious^2^	Undetected	Low
ACM	7 (10137)	Not serious	Not serious (I²=0%)	Serious^1^	Serious (incoherence)^3^	Undetected	Low
MACE	4	Not serious	Not serious (I²=0%)	Serious^1^	Not serious	Undetected	Moderate
HF hospitalization	5	Not serious	Moderate (I²=59%)	Serious^1^	Serious²	Undetected	Low
KCCQ-CSS	3	Not serious	Not serious (I²=0%)	Not serious	Not serious	Undetected	Moderate
6MWD	4	Not serious	Not serious (I²=0%)	Not serious	Not serious	Undetected	Moderate

Composite Outcome Definitions Across Studies

An important source of clinical heterogeneity is the variation in composite outcome definitions across studies. SUMMIT [11] defined its primary endpoint as “CV death or worsening HF event,” which included urgent HF visits and diuretic intensification in addition to HF hospitalization. FIGHT [12] used “death or HF hospitalization” (not restricted to CV death). CVOT subgroups (SELECT HF [26], SOUL HF [27], FLOW HF [28], Harmony Outcomes HF [24], LEADER HF [31], and REWIND HF [32]) used trial-specific definitions that may not align with either definition. These differences in what constitutes an “event” complicate cross-study comparisons and may contribute to the observed heterogeneity. The per-study composite endpoint definitions are mapped in Table 8.

**Table 8:** Composite outcome definitions by study Per-study mapping of primary composite endpoint components and definitional notes, with reference numbers in the Ref column. Each row reports one of the eight studies contributing to the primary composite. CV, cardiovascular; HF, heart failure; CVOT, cardiovascular outcomes trial

Ref	Study	Trial	Primary composite definition	Components	Notes
[11]	Packer (2025)	SUMMIT	CV death or worsening HF event	CV death; first HF hospitalization; urgent HF visit; intensification of diuretics	Broader definition, including outpatient worsening events
[12]	Margulies (2016)	FIGHT	Death or HF hospitalization	All-cause death; HF hospitalization	Includes all-cause (not CV-specific) death
[24]	Ferreira (2022)	Harmony Outcomes (HF post-hoc)	CV death or HF hospitalization	CV death; first HF hospitalization	Post-hoc subgroup of Harmony Outcomes parent CVOT
[26]	Deanfield (2024)	SELECT (HF subgroup)	CV death or HF hospitalization	CV death; first HF hospitalization	Standard CVOT subgroup composite
[27]	Pop-Busui (2026)	SOUL (HF analysis)	CV death or HF hospitalization	CV death; first HF hospitalization	Standard CVOT subgroup composite
[28]	Pratley (2024)	FLOW (HF subgroup)	CV death or HF hospitalization	CV death; first HF hospitalization	Standard CVOT subgroup composite
[31]	Marso (2020)	LEADER (HF subgroup)	CV death or HF hospitalization	CV death; first HF hospitalization	Pre-specified subgroup of LEADER parent CVOT
[32]	Branch (2022)	REWIND (HF post-hoc)	CV death or HF hospitalization	CV death; first HF hospitalization	Post-hoc subgroup of REWIND parent CVOT

Interpretation

This systematic review and meta-analysis provides the most comprehensive synthesis to date of GLP-1 RA effects in HF, incorporating 14 studies encompassing 18,184 patients in HF subgroups or dedicated HF arms, of whom 2499 were directly randomized in six dedicated HF trials, and 1031 (from SUMMIT [11] and FIGHT [12]) contributed adjudicated event counts for the primary composite outcome. The primary composite of CV death and HF hospitalization showed a 14% relative risk reduction (HR 0.86) that did not reach statistical significance (P=0.067). Nevertheless, GLP-1 RAs were associated with significant reductions in ACM (HR 0.86; low-certainty evidence) and MACE (HR 0.80; moderate-certainty evidence), along with clinically meaningful improvements in quality of life (KCCQ-CSS +7.4 points) and functional capacity (6MWD +17.6 m).

The prespecified HKSJ method provides more accurate CI coverage than the DerSimonian-Laird approach when the number of studies is small [17,18], and the moderate between-study heterogeneity (I²=47%) appropriately widened the CI. Notably, the fixed-effect model yielded a statistically significant result (P=0.0006), and excluding the FIGHT trial [12], which enrolled a distinctly different population of patients with acutely decompensated HFrEF, resulted in statistical significance (P=0.011) with markedly reduced heterogeneity (I²=16%).

Heterogeneity and the FIGHT Signal

The FIGHT trial warrants specific discussion. Although FIGHT enrolled acutely decompensated patients, all participants had established chronic HFrEF with an acute exacerbation requiring hospitalization, thereby meeting our inclusion criterion of an established HF diagnosis; prespecified sensitivity analyses excluding this trial were performed to address the resulting clinical heterogeneity. Unlike all other included studies, FIGHT enrolled patients with recent acute HF decompensation and severe HFrEF (mean LVEF 25%), a population with fundamentally different pathophysiology and hemodynamic status [12]. The numerically unfavorable composite HR of 1.30 in FIGHT contrasts sharply with the consistent benefit observed across the other studies. This divergence may reflect the unsuitability of GLP-1 RA initiation during acute HF decompensation, timing-dependent effects, or the distinct mechanisms underlying acute HFrEF versus chronic HFpEF.

The clinical heterogeneity of the included studies deserves additional emphasis and is statistically supported by the significant subgroup interactions by HF phenotype (P-interaction=0.018) and GLP-1 RA agent (P-interaction=0.045). This meta-analysis pools data from populations as disparate as obese patients with chronic HFpEF (SUMMIT [11] and STEP-HFpEF [9]), acutely decompensated HFrEF patients (FIGHT [12]), and diabetes-focused CVOTs in which HF was a secondary classification (LEADER [31], EXSCEL [29,30]). While precedent exists for class-level pooling in HF meta-analyses, sodium-glucose cotransporter-2 (SGLT2) inhibitor meta-analyses similarly combined dedicated HF trials with CVOT HF subgroups, an important distinction is that the SGLT2 inhibitor evidence base included five large dedicated HF trials with standardized composite endpoints and adjudicated events, whereas only two of the eight studies reporting the primary composite outcome (SUMMIT and FIGHT) provided event counts from dedicated HF populations. The 95% PI of 0.64 to 1.16 is perhaps the most informative summary of the expected treatment-effect range, indicating that while most future HF studies of GLP-1 RAs would likely show benefit, a study conducted in an unfavorable population (e.g., acute HFrEF) could plausibly show harm. The phenotype-by-treatment interaction reinforces this caution: the divergent point estimates between HFpEF (HR 0.62) and HFrEF (HR 1.30) are unlikely to reflect chance alone, although confounding among phenotype, agent (tirzepatide vs. liraglutide), and acuity (chronic vs. acute) cannot be disentangled with two single-study strata.

Heart Failure With Preserved Ejection Fraction and Obesity

The signal for benefit appears strongest in HFpEF with concurrent obesity, as supported by the dedicated trials. SUMMIT [11] demonstrated a 38% reduction in the composite of CV death or worsening HF with tirzepatide, while the STEP-HFpEF trials [9,10] showed substantial improvements in symptoms and functional capacity with semaglutide. This is mechanistically plausible, as obesity-related HFpEF is characterized by systemic inflammation, metabolic dysfunction, increased epicardial fat, and volume overload [4].

An important finding is the Harmony Outcomes HF subgroup [24], which showed an HR of 1.06 (95% CI 0.79 to 1.43) for the composite outcome in patients with established HF, contrasting with the significant CV benefit observed in the overall CVOT population. This suggests that the CV benefits of albiglutide in the broader T2DM population may not extend to patients with established HF or, alternatively, that the post hoc subgroup analysis was underpowered to detect a modest effect.

Class Versus Agent Effects

Semaglutide was the most studied agent, contributing five of 14 included studies across both dedicated HF trials and CVOT subgroups. The consistent benefit observed across these studies, spanning different doses (1.0 mg, 2.4 mg, and oral), populations (HFpEF, mixed), and trial designs, strengthens the evidence for a class effect, with semaglutide having the most robust evidence base. Tirzepatide, as a dual GIP/GLP-1 receptor agonist, showed the largest effect in SUMMIT, though the contribution of GIP receptor agonism to HF outcomes remains to be fully elucidated.

Comparison With Competing Meta-Analyses

Our findings should be contextualized alongside recent competing meta-analyses. Behers et al. [13] reported similar pooled estimates for composite outcomes but did not explicitly address the EXSCEL double-counting issue or the divergent mortality signals between CVOT subgroups and dedicated trials. Zhang et al. [14] focused on a narrower set of trials and did not include SUMMIT or SOUL HF data. Our analysis adds the most comprehensive cross-spectrum synthesis to date, explicitly addressing clinical heterogeneity, outcome definition variability, and the critical distinction between indirect (CVOT) and direct (dedicated trial) evidence for mortality.

Mortality: Direct Versus Indirect Evidence

The ACM finding (HR 0.86; I²=0%) warrants nuanced interpretation despite its statistical consistency. The zero heterogeneity reflects the fact that all individual study point estimates fell within a narrow range, but the 95% PI (0.75 to 1.00) reaches the null boundary, underscoring uncertainty about whether a mortality benefit would be reproduced consistently in future HF populations. This pooled signal was driven primarily by CVOT subgroup analyses, where the mortality benefit may reflect the broader CV effects of GLP-1 RAs in patients with diabetes and established CVD rather than HF-specific mechanisms. Importantly, among dedicated HF trials, SUMMIT showed numerically higher ACM with tirzepatide (HR 1.25), and both FIGHT (HR 1.10) and LIVE (HR 1.10) showed similar non-significant trends toward harm. The pooled mortality benefit should therefore be interpreted cautiously: it remains uncertain whether GLP-1 RAs reduce mortality through direct HF mechanisms or through broader cardiometabolic pathways captured in CVOT populations. The GRADE certainty was rated low (downgraded for indirectness due to CVOT subgroups and for incoherence given the divergent signal between CVOT-derived benefit and dedicated trial directional harm).

Limitations

Several limitations should be acknowledged. First, CVOT subgroup analyses introduce indirectness, as HF was not the primary trial endpoint and subgroup analyses may have limited statistical power and, in some cases, a post hoc design. Second, individual patient data were not available, precluding exploration of patient-level predictors of response and introducing the risk of ecological fallacy in subgroup analyses. Third, TSA was limited to studies reporting event counts; most studies reported HRs without corresponding event counts. Fourth, safety data were limited to dedicated HF trials, as CVOTs did not report safety endpoints for HF subgroups. Fifth, the search was restricted to PubMed, Cochrane CENTRAL, and ClinicalTrials.gov; Embase and Web of Science were not searched because no institutional access was available. Sixth, the relatively short follow-up of some trials (12 to 52 weeks) may not capture longer-term effects. Seventh, two EXSCEL subgroup analyses were derived from the same parent trial and included partially overlapping populations [29,30]. To avoid double-counting for secondary outcomes, the primary analysis used only Neves et al. [29] (by LVEF), with sensitivity analyses substituting Fudim et al. or excluding both, yielding directionally consistent results. Eighth, no kappa statistic was calculated because the initial screening reconstruction was not performed as blinded, parallel dual-reviewer screening; this limitation was documented transparently, and all included studies underwent second-reviewer verification. Finally, the substantial weight reduction observed (-9.3 kg) makes it difficult to disentangle the direct cardiac effects of GLP-1 RAs from the indirect benefits of weight loss. This review was conducted without institutional resources, including library support, institutional review board oversight, or external biostatistical review.

Clinical implications

These findings support the emerging role of GLP-1 RAs in the management of HF, particularly in patients with HFpEF and obesity, a population with limited therapeutic options. The 2023 European Society of Cardiology focused update already acknowledged the potential of semaglutide in obese HFpEF patients, and our meta-analysis provides quantitative evidence supporting this position [33]. The strongest evidence base supports use in patients with HFpEF and concurrent obesity (BMI ≥30 kg/m²), in whom the dedicated trials (SUMMIT [11], STEP-HFpEF [9], STEP-HFpEF-DM [10]) consistently demonstrated improvements in HF events, symptoms, and functional capacity. For HFrEF, the evidence remains inconclusive and suggests caution, particularly in the acute decompensated setting, where the FIGHT trial signal of a directionally unfavorable effect [12] cannot be dismissed. Practical considerations include the need for gradual dose titration to mitigate gastrointestinal adverse events, monitoring for treatment discontinuation (which trended numerically higher with GLP-1 RAs in our safety analysis), and ongoing access and cost barriers in resource-limited settings. In HF populations specifically, where polypharmacy and chronic disease management already burden adherence, the numerically higher treatment-discontinuation signal warrants explicit pretreatment counseling and dose-titration protocols to preserve long-term GLP-1 RA exposure. The significant ACM reduction is a compelling finding that, if confirmed in future dedicated HF mortality trials, could substantially expand the treatment paradigm for HF.

Future research should prioritize: (1) dedicated HF mortality trials of GLP-1 RAs using adjudicated endpoints and prespecified subgroups by HF phenotype and concomitant SGLT2 inhibitor use; (2) head-to-head and combination studies with SGLT2 inhibitors to clarify additive versus overlapping benefit; (3) longer-term safety data extending beyond the 12 to 52 weeks of follow-up that characterized limited included trials; (4) mechanistic substudies disentangling the contribution of weight loss from direct cardiac effects; and (5) trials in HFrEF with chronic stable disease (rather than acute decompensation) to determine whether the FIGHT signal generalizes or reflects timing-specific effects.

## Conclusions

In adults with HF across the ejection-fraction spectrum, GLP-1 RAs did not significantly reduce the primary composite outcome of CV death and HF hospitalization, although the direction of effect favored treatment. GLP-1 RAs were associated with significant reductions in ACM and MACE, but the mortality benefit was driven principally by CVOT subgroup analyses, whereas dedicated HF trials showed directional harm; the certainty of evidence for ACM was therefore rated as low. Clinically meaningful improvements in quality of life and functional capacity were consistently observed in dedicated trials enrolling patients with HFpEF and obesity. Effects on the primary composite outcome differed significantly by HF phenotype and GLP-1 RA agent, supporting a phenotype-stratified rather than class-uniform interpretation. The strongest current evidence supports the use of GLP-1 RAs in HFpEF with obesity. The signal in HFrEF, particularly in acutely decompensated HFrEF, remains uncertain and warrants caution. Dedicated HF mortality trials with adjudicated endpoints are needed before the pooled mortality reduction can be interpreted as evidence of a true HF-specific benefit.

## Appendices

Appendix A: database sensitivity (EMBASE omission rationale)

The systematic review searched PubMed (including MEDLINE), Cochrane CENTRAL, and ClinicalTrials.gov. Embase and Web of Science were not searched because no institutional access was available to either. We assessed the likely impact of this database limitation by: 1. Cross-checking each included trial against Embase via the trial's clinical trial registry record (NCT identifier, available for all 14 included studies). All 14 included studies were identifiable in PubMed/MEDLINE coverage; no candidate trial was identified through Embase-only indexing. 2. Comparing the reference lists of two recent overlapping meta-analyses (Behers et al. 2026 [13] and Zhang et al. 2026 [14]), which did include Embase, against our own. No additional eligible trials appeared in their lists that were not already captured by our PubMed/Cochrane CENTRAL/ClinicalTrials.gov search. 3. Quantifying the pre-specified eligible population: dedicated heart failure (HF) trials of glucagon-like peptide-1 receptor agonists (GLP-1 RAs) and HF subgroup analyses of cardiovascular outcomes trials (CVOTs) are uniformly published in PubMed-indexed cardiology, internal medicine, and diabetes/endocrinology journals; no eligible publication is restricted to Embase- or Web-of-Science-only journals.

For these reasons, we judge the Embase omission unlikely to have materially altered the pooled estimates. We acknowledge this database limitation under Limitations (Review section, Interpretation) and as Limitation #5 in the limitations enumeration.

Appendix B: screening algorithm and dual-reviewer verification

Initial title and abstract screening was performed using algorithmic filters (Python pipeline) rather than blinded, parallel dual-reviewer screening. The pipeline applied three sequential filters: 1. Domain filter exclude records whose Medical Subject Headings (MeSH) descriptors or title/abstract clearly placed them outside the population, intervention, comparator, outcome (PICO) framework - animal studies, in vitro studies, single-arm or non-randomized studies, non-HF populations, and non-GLP-1 RA interventions. 2. Eligibility filter includes records whose title or abstract referenced HF (or any HF phenotype synonym) and any of the seven eligible GLP-1 RAs (semaglutide, liraglutide, tirzepatide, dulaglutide, exenatide, lixisenatide, albiglutide). 3. Trial design filter keeps records identifiable as randomized controlled trials (publication type tags or explicit mentions of randomization) and excludes conference abstracts without full publications, case reports/series, and editorials.

The 200 records that passed the algorithmic pipeline then underwent full PICO eligibility assessment with second-reviewer verification of every eligibility decision and every included study, including manual reconciliation of borderline cases. Because the initial exclusion stage was not performed as blinded, parallel dual-reviewer screening, no inter-rater agreement statistic (Cohen's kappa) was calculated for the title/abstract phase. We note this transparently as a methodological limitation. The downstream full-text and inclusion stages were independently verified by both reviewers, and all 14 included studies were reconciled to consensus.

This transparency-first design is documented in International Prospective Register of Systematic Reviews (PROSPERO) Amendment 3 (filed before journal submission) and in the Amendments Log of the Supplementary Material associated with the Zenodo deposit.

Appendix C: sensitivity analyses beyond the primary composite

In addition to the pre-specified sensitivity analyses for the primary composite (fixed-effect, exclusion of FIGHT, exclusion of tirzepatide, dedicated trials only, leave-one-out [LOO]), we performed three sensitivity analyses for all-cause mortality (ACM), major adverse cardiovascular events (MACE), and HF hospitalization that addressed the EXSCEL double-counting issue: 1. Primary (used in the main analyses): Neves et al. 2025 [29], stratified by left ventricular ejection fraction (LVEF), as the LVEF-based stratification more closely matches the PICO framework. 2. Sensitivity 1 (Fudim instead of Neves): substituting Fudim et al. 2020 [30], stratified by HF status. ACM hazard ratio (HR) 0.88 (95% confidence interval [CI] 0.81 to 0.95); MACE HR 0.82 (95% CI 0.69 to 0.99); HF hospitalization HR 0.88 (95% CI 0.63 to 1.24). 3. Sensitivity 2 (excluding both EXSCEL subgroups): ACM HR 0.88 (95% CI 0.78 to 0.98); MACE HR 0.78 (95% CI 0.64 to 0.95); HF hospitalization HR 0.86 (95% CI 0.51 to 1.46).

All three approaches yielded directionally consistent results, supporting the robustness of the secondary outcome estimates.

Appendix D: data, code, and protocol availability

Protocol Registration

PROSPERO CRD420261299844 (registered 03/02/2026; with Amendments 1, 2, and 3 filed publicly). URL: https://www.crd.york.ac.uk/prospero/display_record.php?RecordID=1299844

Preprint

medRxiv Digital Object Identifier (DOI): 10.64898/2026.02.10.26345946 (latest version reflects this manuscript revision).

Data and Code Archive

All raw data, analysis code, intermediate outputs, figures, tables, and the Preferred Reporting Items for Systematic Reviews and Meta-Analyses (PRISMA) 2020 checklist are publicly archived in a versioned Zenodo deposit:

Concept Digital Object Identifier

The concept DOI, which always resolves to the latest version, is 10.5281/zenodo.18580111.

This manuscript corresponds to Zenodo version 3 (published May 2, 2026): 10.5281/zenodo.19981392. The Zenodo deposit and GitHub repository contain the complete R analysis pipeline (scripts/R/), including scripts for the primary, secondary, sensitivity, subgroup, publication-bias, Trial Sequential Analysis (TSA), and Grading of Recommendations, Assessment, Development, and Evaluations (GRADE) analyses.

The Zenodo deposit and GitHub repository contain the Python search and screening pipeline (scripts/python/), raw search exports (deidentified, query-only; no patient-level data), the Phase 4 extraction form (data/phase4_extraction/meta_analysis_input.csv), Phase 5 analysis-ready data and result tables (data/phase5_analysis/), manuscript source files (.Rmd, .docx, and .pdf), and the PRISMA 2020 checklist.

Reproducibility

The full pipeline can be re-executed via Rscript scripts/R/run_all.R (R version 4.4 or later). Package versions are pinned via the renv lockfile included in the GitHub repository. The complete analysis takes approximately 4-6 minutes on a modern workstation.
